# Aloe-Emodin-Mediated Photodynamic Therapy Attenuates Sepsis-Associated Toxins in Selected Gram-Positive Bacteria In Vitro

**DOI:** 10.4014/jmb.2105.05024

**Published:** 2021-07-23

**Authors:** Woodvine Otieno, Chengcheng Liu, Yanhong Ji

**Affiliations:** Department of Pathogenic Microbiology and Immunology, School of Basic Medical Sciences, Xi'an Jiaotong University Health Science Center, 76 West Yanta Road, Xi'an 710061, P.R. China

**Keywords:** Aloe-emodin, cytolysin, δ-hemolysin, gram-positive bacteria, pneumolysin, sepsis

## Abstract

Sepsis is an acute inflammatory response that leads to life-threatening complications if not quickly and adequately treated. Cytolysin, hemolysin, and pneumolysin are toxins produced by gram-positive bacteria and are responsible for resistance to antimicrobial drugs, cause virulence and lead to sepsis. This work assessed the effects of aloe-emodin (AE) and photodynamic therapy (PDT) on sepsis-associated gram-positive bacterial toxins. Standard and antibiotic-resistant *Enterococcus faecalis*, *Staphylococcus aureus*, and *Streptococcus pneumonia* bacterial strains were cultured in the dark with varying AE concentrations and later irradiated with 72 J/cm^-2^ light. Colony and biofilm formation was determined. CCK-8, Griess reagent reaction, and ELISA assays were done on bacteria-infected RAW264.7 cells to determine the cell viability, NO, and IL-1β and IL-6 pro-inflammatory cytokines responses, respectively. Hemolysis and western blot assays were done to determine the effect of treatment on hemolysis activity and sepsis-associated toxins expressions. AE-mediated PDT reduced bacterial survival in a dose-dependent manner with 32 μg/ml of AE almost eliminating their survival. Cell proliferation, NO, IL-1β, and IL-6 cytokines production were also significantly downregulated. Further, the hemolytic activities and expressions of cytolysin, hemolysin, and pneumolysin were significantly reduced following AE-mediated PDT. In conclusion, combined use of AE and light (435 ± 10 nm) inactivates MRSA, *S. aureus* (ATCC 29213), *S. pneumoniae* (ATCC 49619), MDR-*S. pneumoniae*, *E. faecalis* (ATCC 29212), and VRE (ATCC 51299) in an AE-dose dependent manner. AE and light are also effective in reducing biofilm formations, suppressing pro-inflammatory cytokines, hemolytic activities, and inhibiting the expressions of toxins that cause sepsis.

## Introduction

Sepsis is an acute inflammatory response towards an infectious pathogen, accompanied by a complex chemical and cellular interaction cascade [1]. This immune response aims to eliminate the invasive microorganisms from the body. Despite the protective cellular mechanism, the acute response may damage tissues, causing life-threatening problems if not quickly and adequately treated [2]. In recent years, sepsis incidence has been rising due to the growing elderly population, prolonged hospitalization, and increasingly aggressive medical procedures; thus, sepsis is causing a significant epidemiological and public health concern [3]. Nosocomial infectious diseases resulting from contaminated blood products, intravenous fluid, and medications have been reported [4]. The sepsis mortality rates vary from 12.8% (sepsis) and 20.7% (severe sepsis) to 45.7% (septic shock) [5].

Gram-negative bacteria lipopolysaccharide (LPS) has been reported as the primary molecule accelerating sepsis by stimulating the release of endogenous mediators, thus causing the pathophysiological changes responsible for high mortality [6]. However, according to recent clinical reports, sepsis arising from gram-positive bacteria has been increasing and is responsible for 50% of septic shock or severe sepsis in intensive care units (ICUs) [7]. Methicillin-resistant *Staphylococcus aureus* (MRSA), *Staphylococcus aureus*, *Streptococcus pneumoniae*, *Enterococcus faecalis*, and vancomycin-resistant *Enterococcus faecalis* (VRE) are among these gram-positive bacteria responsible for sepsis.

In establishing infections, pathogens employ various virulence factors protecting them from the host's innate immune system and enabling them to invade the mucosal barriers, spread, and multiply in the neighboring organs. These gram-positive bacteria each express various exotoxins, consequently conferring antibiotic resistance. To counteract resistance to antimicrobials and to contain immune evasion by exotoxins, a search for alternative therapy is necessary.

A promising option is photodynamic therapy (PDT). The procedure involves combining appropriate light wavelengths and a photosensitizer (PS) dye sensitive to light. The irradiation generates reactive oxygen species (ROS), leading to the destruction of biomolecules and killing disease-causing bacterial cells. Compared to other ways of treatment, the advantages of PDT are outstanding. The PS mainly demonstrates minimal dark cytotoxicity and only turns highly cytotoxic on irradiation with light [8]. The treatment region size is dependent on the area of light irradiation, hence ensuring minimum harmful effect on the adjacent normal tissues. The technique is generally based on the transfer of energy from excited PSs to molecular oxygen to generate singlet oxygen (1O_2_), which instantly destroys bacterial biomolecules and initiates the death of cells.

**Fig. 1:**
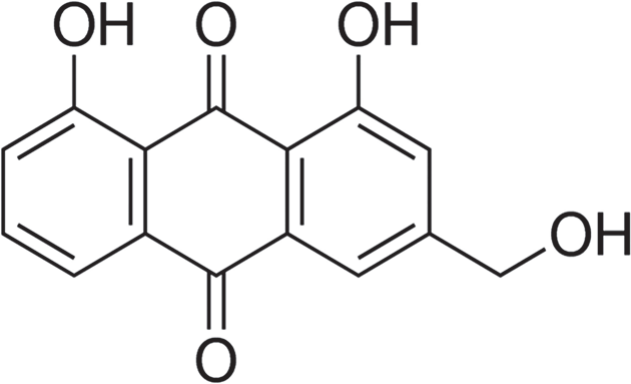
The chemical structure of aloe-emodin.

Aloe-emodin (1,8-dihydroxy-3-(hydroxymethyl)anthraquinone) (Fig. 1) is a naturally occurring anthraquinone isolated from *Aloe barbadensis* Miller, and *Rheum palmatum* L. AE has been shown to have anti-cancer, anti-inflammatory, antiviral, and antiparasitic effects [9]. Our previous report also demonstrated the anti-fungal activity of AE against drug-sensitive and drug-resistant *Candida albicans* [10]. Because of its ability to exhibit fluorescence and its maximum excitation wavelength of 430 nm, AE is a suitable PS [11]. The maximum peak of absorption in the blue region also makes AE a promising PS for treatment of superficial infections [12]. However, the role of AE-mediated PDT on sepsis-related toxins remains unclear. This work assessed the AE-mediated PDT on Standard and antibiotic-resistant *S. aureus* (MRSA), *S. pneumoniae*, *E. faecalis*, and vancomycin-resistant *E. faecalis* (VRE) gram-positive bacteria.

## Materials and Methods

### Photosensitizer (PS) and Light

AE (Nanjing Jinzhu Biotechnology Co., China) was freshly prepared by dissolving in dimethyl sulfoxide (DMSO) (Sigma-Aldrich) to prepare working concentrations of 2, 4, 8, 16, and 32 μg/ml for various assays, as previously reported [13], after filtration using 0.22 μM sterile filters. The AE absorption spectrum was recorded on a UV-Vis spectrophotometer (Agilent 8453, USA). A 50W xenon lamp (Ceaulight CELHXF300, China) was used for irradiation assays. White light with a 435 ± 10 nm wavelength was determined by an optical filter (Ceaulight CEL UVIRCUT PD-145, China) and used for assays. The Xenon lamp emission spectrum was recorded on a fiber optic spectrometer (S3000, Seemantech, China). A power meter (Ceaulight CEL-NP2000, China) was used for adjustment of the power density of 0.08 W/cm^2^ at the sample level. The distance between the optical filter and the bacterial samples was 10 cm. To ensure constant cooling, an ice-cold water filter (1 cm in width) was placed between the optical filter and the experimental samples. The samples were irradiated using 435± 10 nm white light for 10 minutes, with 72 J/cm^2^ energy density.

### Bacterial Strains and Culture Conditions

Standard *S. aureus* (ATCC 29213), *S. pneumoniae* (ATCC 49619), and *E. faecalis* (ATCC 29212) were all stored in our experimental laboratory. Clinical isolates of VRE (ATCC 51299), MRSA, and MDR *S. pneumoniae* (ATCC 49619) were obtained from the First Affiliated Hospital of Xi'an Jiaotong University, in Xi'an, China.

Standard and antibiotic-resistant *S. aureus* and *E. faecalis* strains were cultured on tryptone soy agar (TSA)(Qingdao Rishui Biotech) for 36 h at 37°C. The resulting colonies were transferred to 10 ml tryptone soy broth (TSB) (Qingdao Rishui Biotech, China) and grown to a log phase. The bacterial samples were then transferred into a 15 ml tube, centrifuged for 15 min at 4,000 × *g*, and washed twice in sterilized PBS. The samples were then diluted to 1 × 10^7^ colony forming units (CFU) per milliliter for subsequent assays. Antibiotic-resistant and standard *S. pneumoniae* bacterial strains were cultivated on blood agar plates (Beijing Kangqiao, China) for 36 h at 37°C. The grown colonies were transferred to 10 ml TSB supplemented in 5% sheep blood. Later, the samples were incubated for 24 h at 37°C with shaking (100 rpm) to a log phase of growth. Pellets were then harvested by centrifugation, washed two times in PBS, and resuspended to 1 × 10^7^ CFU/ml density for subsequent assays.

### Bacterial PDI in Planktonic Culture

Approximately 1 × 10^7^ CFU/ml of bacteria in 2 ml suspension were centrifuged for 15 min at 4,000 × *g* and resuspended in 2 ml AE with various concentrations (0, 2, 4, 8, 16, 32 μg/ml). Samples were then incubated in the dark for 30 min, then transferred to 35-mm polystyrene culture dishes (Corning, USA). Irradiation was then done for 900 s using 435 ± 10 nm light. Later, the pellets were spin for 10 min at 4,000 rpm, resuspended using sterilized PBS, and followed by a 10-fold serial dilution. For standard and antibiotic-resistant *S. aureus* and *E. faecalis* strains, 20 μl of dilution was spread in triplicate on TSA. Approximately 20 μl of *S. pneumoniae* dilution was also spread on blood agar plates in triplicate and cultured for 48 h at 37°C. The grown colonies were counted, and bacterial survival was determined using the following equation: *N_PDI_*/*N_0_*, where *N_PDI_* (CFU/ml post-PDI) and *N_0_* (number of CFU/ml without treatment).

### Biofilm Formation Assay

Biofilm formation was assessed through the tissue culture plate method. Suspensions of bacteria were diluted in sterilized TSB to 1 × 10^6^ CFU/ml, and 200 μl of the suspensions was inoculated into 96-well microplates and cultured for 24 h at 37°C. After discarding the medium, the biofilms were washed in PBS two times. The biofilm fixation was done for 10 min using 95% ethanol and stained for 15 min with crystal violet (0.1 % (w/v) (200 μl) at room temperature. The wells were then washed two times with PBS, dried for 2 h at 37°C, and the formation of biofilm quantified through crystal violet stain solubilization in 30% (w/v) glacial acetic acid (200 μl) by shaking at 200 rpm for 10 min. The absorbance was finally determined at 492 nm (A492nm) using a microplate reader (Thermo Fisher 1510, Finland). A492nm > 0.240 was used to indicate the formation of biofilm.

### Bacterial Biofilms PDI

Approximately 1 × 10^6^ CFU/ml of the samples in 2 ml suspension was inoculated into 24-well microplates containing sterilized glass coverslips and incubated for 24 h at 37°C. After discarding the culture medium, biofilms adhered to coverslips were carefully washed twice with sterile PBS. The coverslips were then transferred to wells of another 24-well microplate pre-filled with 2 ml of AE at a concentration of 32 μg/ml. The microplate was later incubated for 30 min at 37°C in the dark. The coverslips were then irradiated for 900 s, gently washed twice, and transferred into the wells of a new 24-well microplate. The biofilms were later resuspended in sterilized PBS (2 ml) and ultrasonicated in a sonicator (Hangzhou Front Ultrasonic FRQ-1002 T, China) for 10 min to dislodge the cells. Samples were then rapidly vortexed for 1 min using a vortex mixer (Haimen Qilinbeier QL-901, China). After a 10-fold serial dilution of the resulting suspensions in sterilized PBS, 20 μl of each bacterial sample was plated in triplicate on TSA. After 24 h of incubation at 37°C, the colonies were counted, and bacterial survival determined as described earlier.

### Cell Culture and Infection

RAW264.7 cells, obtained from the institution biobank, were first cultivated in T-25 flasks in DMEM augmented with 10% FBS to approximately 75% confluence. The cells were then seeded in 6-well plates and infected with various gram-positive bacteria; MRSA, *S. aureus*, *E. faecalis*, VRE, *S. pneumoniae*, and MDR *S. pneumoniae* for 1 h at 10 multiplicity of infection (MOI) [14]. For longer incubation time, the RAW264.7 cells were washed three times in PBS to eliminate unbound infectious bacteria and incubated further with a complete media supplemented with 16 μg/ml of vancomycin and 150 μg/ml of gentamicin to inactivate extracellular bacteria. After 12 h, AE (32 μg/ml) in complete fresh media was added to the cells and incubated for 30 min. Later, cells were irradiated for 900 s and then incubated further for 12 h at 37°C. For negative control, RAW264.7 cells uninfected with the study gram-positive bacteria were treated similarly to the experimental groups in every aspect.

### Cell Proliferation Assay

RAW264.7 cell survival was determined as described previously [14]. Summarily, approximately 10^6^ cells/well were plated into 6-well plates and incubated for 24 h at 37°C and 5% CO_2_ before infection. Triplicate wells of RAW264.7 cells were infected with MRSA, *S. aureus*, *E. faecalis*, VRE, *S. pneumoniae*, MDR *S. pneumoniae* at an MOI of 10 at 37°C and 5% CO_2_ for 1 h. The cells were later washed thrice in PBS and incubated further with DMEM augmented with 10% FBS, 16 μg/ml vancomycin, and 150 μg/ml gentamicin. Cells were then incubated with 32 μg/ml of AE for 30 min, irradiated (72 J/cm^2^), and incubated for 48 h at 37°C in the dark. AE cytotoxicity was determined with Cell Counting Kit-8 (CCK-8) (Solarbio, China), following the manufacturer's guidelines. Summarily, CCK-8 solution (10 μl) was added to every well, and the plates were subsequently incubated for 1 h at 37°C. The absorbance was read in a microplate reader at 450 nm. The rate of cell survival was calculated.

### Assessment of Nitric Oxide

Nitric oxide (NO) generated by bacterial infected cells was measured using Griess reagent [15]. The bacteria-infected RAW264.7 cells were plated in 24-well culture plates at 5 × 10^5^ cells/ml, incubated at 37°C overnight, and treated with AE (32 μg/ml ) for 30 min. The cells were then irradiated for 900 s and incubated further for 24 h. Later, cell supernatants (50 μl) from every well were obtained and added in 96-well plates. Equal amounts of commercially acquired Griess reagent (Beyotime) were added as per the manufacturer’s protocol. The plates were finally incubated at room temperature. Sodium nitrite reagent was used as the standard. The reaction was determined on a microplate reader (Thermo Fisher 1510) at 540 nm OD. Data were reported as the mean values from assays done in triplicate.

### Cytokine Analysis

Approximately 5 × 10^5^ cells/ml of RAW264.7 cells, infected with various study bacteria, were seeded in 24-well culture plates and incubated at 37°C overnight. Proliferated cells were then treated with 32 μg/ml of AE, incubated for 30 min, irradiated for 900 s, and further incubated for 24 h. The supernatant free of cells was collected to determine the concentration of IL-1β and IL-6 through enzyme-linked immunosorbent assay (ELISA), following the manufacturer's guidelines (R&D Systems, USA). The experiments were carried out in triplicates.

### Hemolysis Assay

The hemolysis studies were undertaken to assess the inhibitory effects of gram-positive bacteria on the hemolytic activity of cytolysin, δ-hemolysin, and pneumolysin toxins after AE-mediated PDT. For this assay, the study was designed into four categories. Each sample of bacteria strain was divided into four groups as follows: group 1 was treated with neither light nor AE (P-L-), group 2 was to be treated with 72 J/cm^2^ of light only (P-L+), group 3 with only 32 μg/ml of AE (P+L-), while group 4 was to be treated with both AE (32 μg/ml) and light (P+L+). According to the procedure, the supernatants of *S. aureus* (ATCC 29213) and MRSA, *S. pneumoniae* (ATCC 49619) and MDR *S. pneumoniae* (ATCC 49619), *E. faecalis* (ATCC 29212) and VRE (ATCC 51299) were first harvested by high-speed spinning at 10,000 × *g* at 4°C for 5 min. Later, 100 μl of each bacterial supernatant was added to 1 ml of sterile PBS buffer. These mixtures (for groups 3 and 4) were pre-incubated with 32 μg/ml of AE for 30 min at 37°C in the dark. Next, the samples were transferred to 6-well plates and irradiated for 900 s (except group 1) and collected back into 15 ml tubes.

Later, 25 μl (5 × 10^6^ cells/ml) of rabbit erythrocytes (R403-0100, Rockland Immunochemicals, Inc, USA) was added to each tube in all the groups and further incubated at 37°C for 30 min. The tubes were centrifuged for 1 min at 6,000 × *g* to remove the unlysed erythrocytes. Hemolytic activity was determined by assessing the supernatant's OD543 values. For the negative control, rabbit erythrocytes (25 μl) were added into deionized water (975 μl), and the bacterial supernatants were used as the 100% hemolysis control. The percentage hemolysis of light-only, AE-only, or AE-PDT samples was determined by comparison of the OD543 values of the test samples and control cultures.

### Western Blot Analysis

For western blot assay, bacterial strains were cultivated in TSB while shaking at 200 rpm at 37°C to an OD_600_ of 0.3. Next, each bacterial culture was divided equally into four 50-ml flasks (P-L-), (P-L+), P+L-) and (P+L+). The P+L- and P+L+ were treated with 32 μg/ml AE and cultured with shaking at 200 rpm in a 37°C incubator for 30 min. Later, the cells (P-L+), (P+L+) were irradiated for 900 s. All the cells were then incubated again until OD_600_ of 2.5 was achieved. After this post-exponential phase of growth, the bacterial cultures were centrifuged for 5 min at 10,000 × *g*. The sample supernatants were heated in Laemmli buffer and loaded on a 12% sodium dodecyl sulfate-polyacrylamide (SDS). δ-hemolysin was separated using 20% SDS. The proteins were then transferred to polyvinylidene fluoride (PVDF) membranes (Millipore, USA). The membrane blocking was done using 5% bovine serum albumin in sterile PBS at 37°C for 2 h. Next, rabbit antibody against cytolysin (anti-PRF1; Cat# SAB1406291-Sigma-Aldrich), δ-hemolysin (Rabbit Anti-S. Aureus delta hemolysin-Cat # SAB1305744; Sigma-Aldrich), and anti-mouse pneumolysin (IF11; sc-80500-Santa Cruz) were diluted to 1:1,000; and incubated with membranes at 4°C overnight. After three washes with PBS-tween, the membranes were incubated with relevant secondary antibodies (1:5,000) conjugated with horseradish peroxidase for 1 h at room temperature in the dark. The bands were detected using Immobilon Western Chemiluminescent HRP substrate (Millipore) and a Chemiluminescent Imaging System. Image J software was used to quantify the band formation after three independent experiments.

### Statistical Analysis

The data were statistically analyzed using GraphPad Prism 7.0 software. The results were presented as the means ± SD of values from three different experiments. The difference between the treated and control groups were analyzed using Student’s *t*-test. Data comparison was done using one-way, or two-way ANOVA where appropriate, followed with a Bonferroni post-hoc test. *p* < 0.01 was considered significant.

## Results

**Fig. 2:**
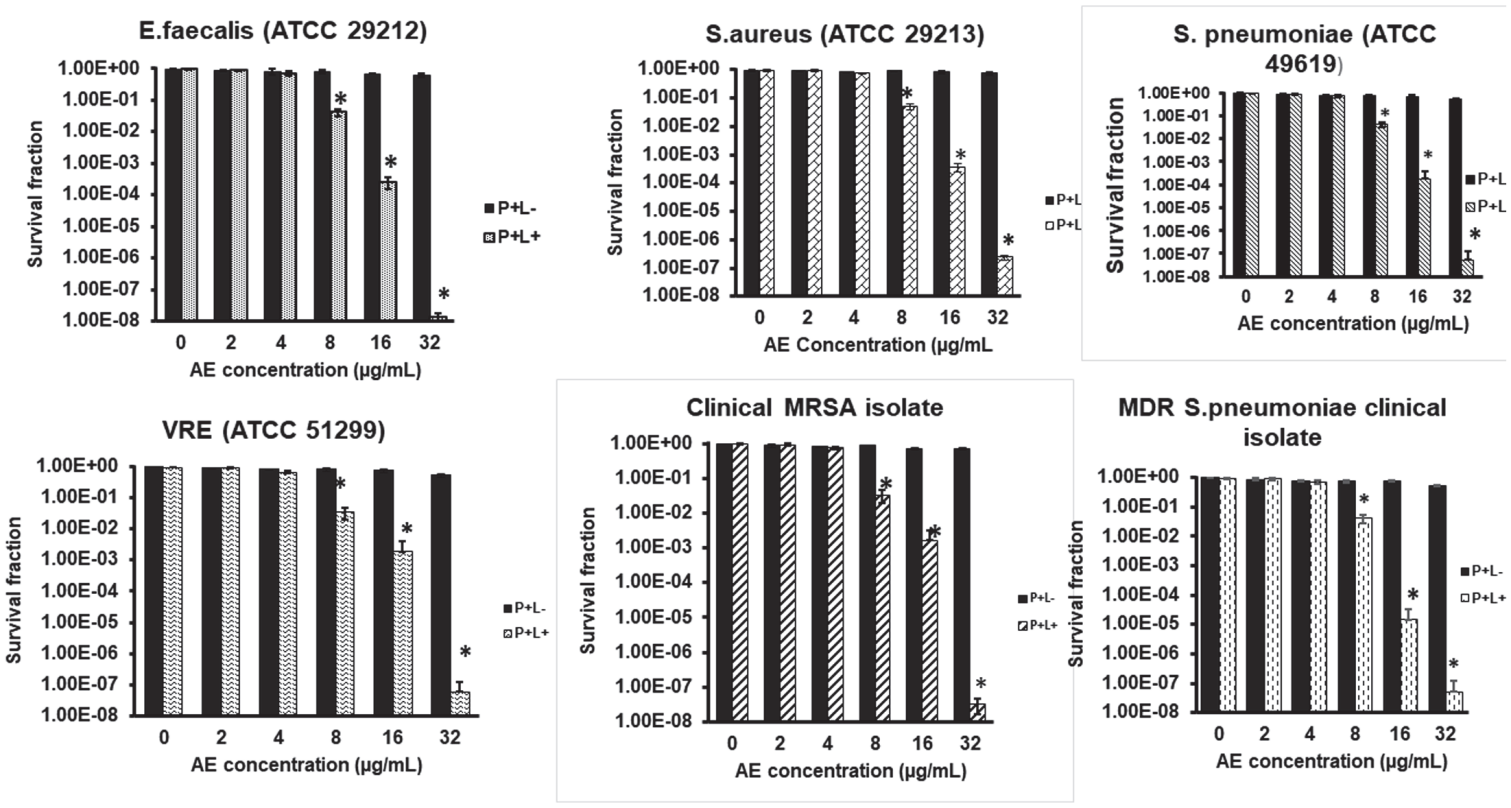
Gram-positive bacteria survival fraction following treatments with various concentrations of AE. P+L- indicates bacterial survival fraction following incubation without light therapy (dark toxicity). P+L+ indicates bacterial survival fraction post-irradiation with 72 J/cm^2^ energy (900 sec). * shows the level of significance at *p* < 0.05 when compared to the untreated controls.

AE demonstrated insignificant dark toxicity at the various concentrations assessed, as shown in Fig. 2. Irradiation without AE also indicated insignificant cytotoxicity (data not shown). AE and light combination demonstrated a bacterial inactivation effect in an AE-dose dependent manner, but only from 8-32 μg/ml. Use of 72 J/cm^2^ of light and 2 to 4 μg/ml of AE did not yield any noticeable bacterial survival reduction, while the use of 8, 16 μg/ml AE resulted in bacterial survival reductions of 1.5 and 3.4 log10 for *E. faecalis* (ATCC 29212) and 1.4 log10 and 2.3 log10 for VRE (ATCC 51299). Similarly, 8 μg/ml and 16 μg/ml AE yielded 1.3 log10 and 3.3 log10 survival reduction in *S. aureus* (ATCC 29213) and 1.3 log10 and 2.6 log10 reduction in the clinical MRSA strain, respectively. For the *S. pneumoniae* (ATCC 49619) standard strain, the treatments yielded a result of 1.3 and 3.2 log10 and 1.4 log10 and 4.9 log10 for the clinical MDR S. pneumonia isolate following the photodynamic treatment with 8 and 16 μg/ml AE, respectively. In all the experimental bacterial strains, the use of 32 μg/ml yielded approximately over 7 log10 reduction in survival, indicating an effective killing.

**Fig. 3:**
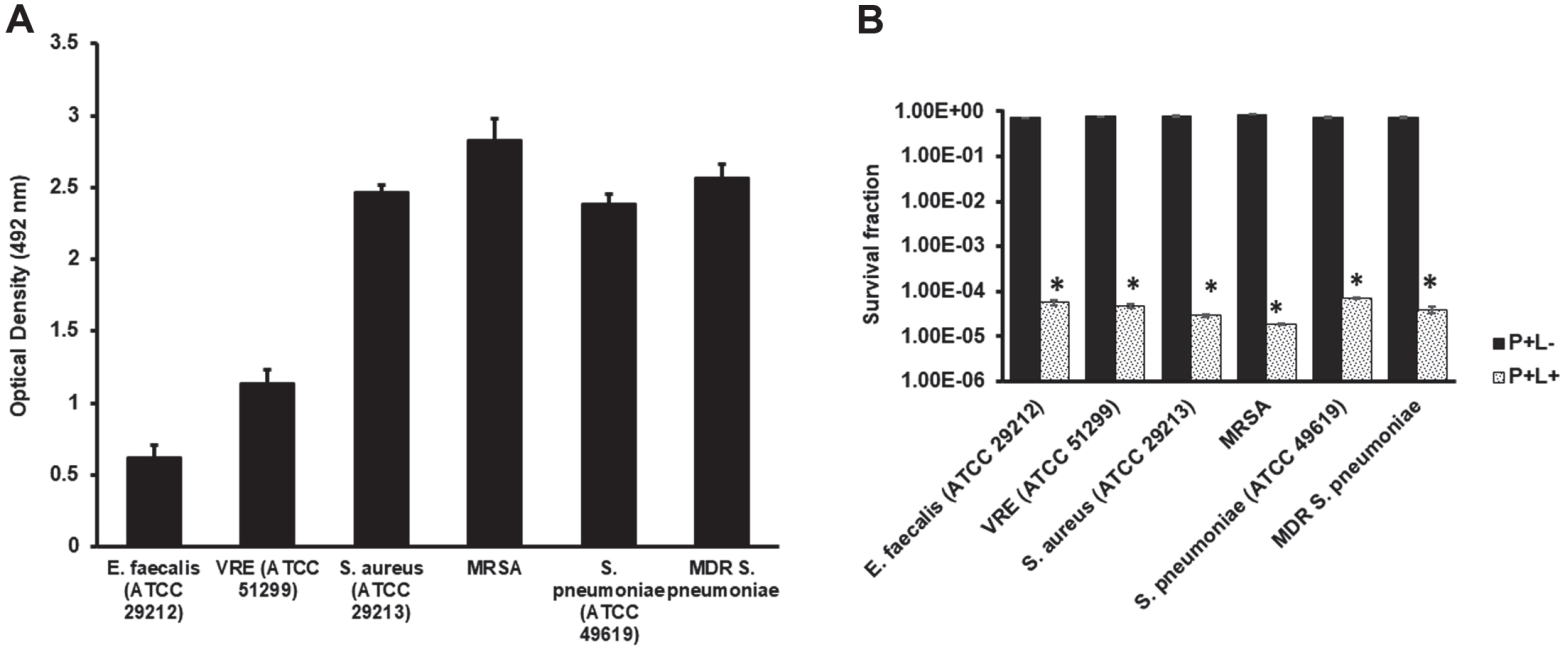
(**A**) Gram-positive antimicrobial-resistant bacteria biofilm formation assays. (**B**) The survival fractions of the bacterial biofilms following treatments with 32 μg/ml of AE and irradiation. P+L- indicates the survival fraction following irradiation with light for 900 sec at a light density of 72 J/cm^2^. *Indicates a statistical significance (*p* < 0.05) when compared to the untreated group.

**Fig. 4:**
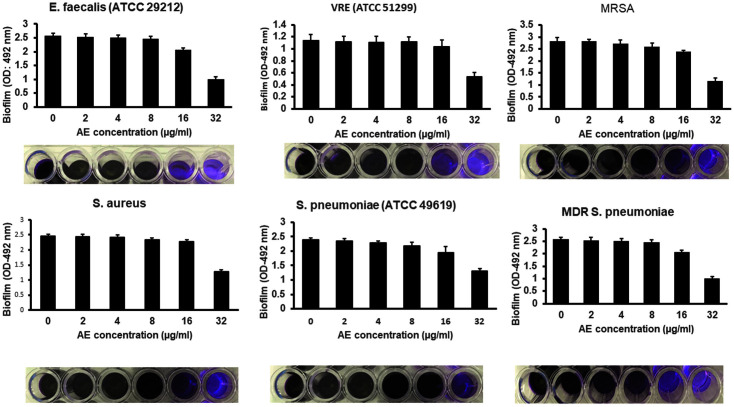
Effects of AE-mediated PDT on gram-positive bacteria biofilm formations. Various stated experimental gram-positive antimicrobial-resistant bacteria were treated with AE (0, 2, 4, 8, 16, or 32) μg/ml and irradiated with 72 J/cm^2^ light. Biofilm formation (OD 492) of various bacteria in 96-well culture plates was quantified after 24 h.

Our observations on the biofilm indicated a significant formation. *E. faecalis* (ATCC 29212) and VRE (ATCC 51299) indicated an OD of 0.622 and 1.032, respectively, *S. aureus* (ATCC 29213) and MRSA yielded 2.466 and 2.825 (Fig. 3A). In *S. pneumoniae* and MDR *S. pneumoniae*, the observations were 2.378 and 2.568 OD, respectively. The observed OD values exceeded the A492 nm 0.240 limit, which indicated gram-positive biofilm-generating bacteria. Further treatment of the produced biofilms with 32 μg/ml AE without irradiation using 72 J/cm^2^ light did not demonstrate a significant reduction in survival following biofilm treatment in the dark, as shown in Fig. 3B. However, the combination of AE and irradiation yielded 4.2 log10 (*E. faecalis*), 3.6 log10 (VRE), 4.3 log10 (*S. aureus*), 4.6 log10 (MRSA), 4.1 log10 (*S. pneumoniae*), and 4.3 log10 (MDR *S. pneumoniae*), as shown in Fig. 3B. Further analysis showed that AE (2, 4, 8, and 16 μg/ml) and irradiation did not affect biofilm reduction. However, the use of 32 μg/ml AE and light significantly regulated the biofilm formation in all the experimental bacterial strains, as shown in Fig. 4.

**Fig. 5:**
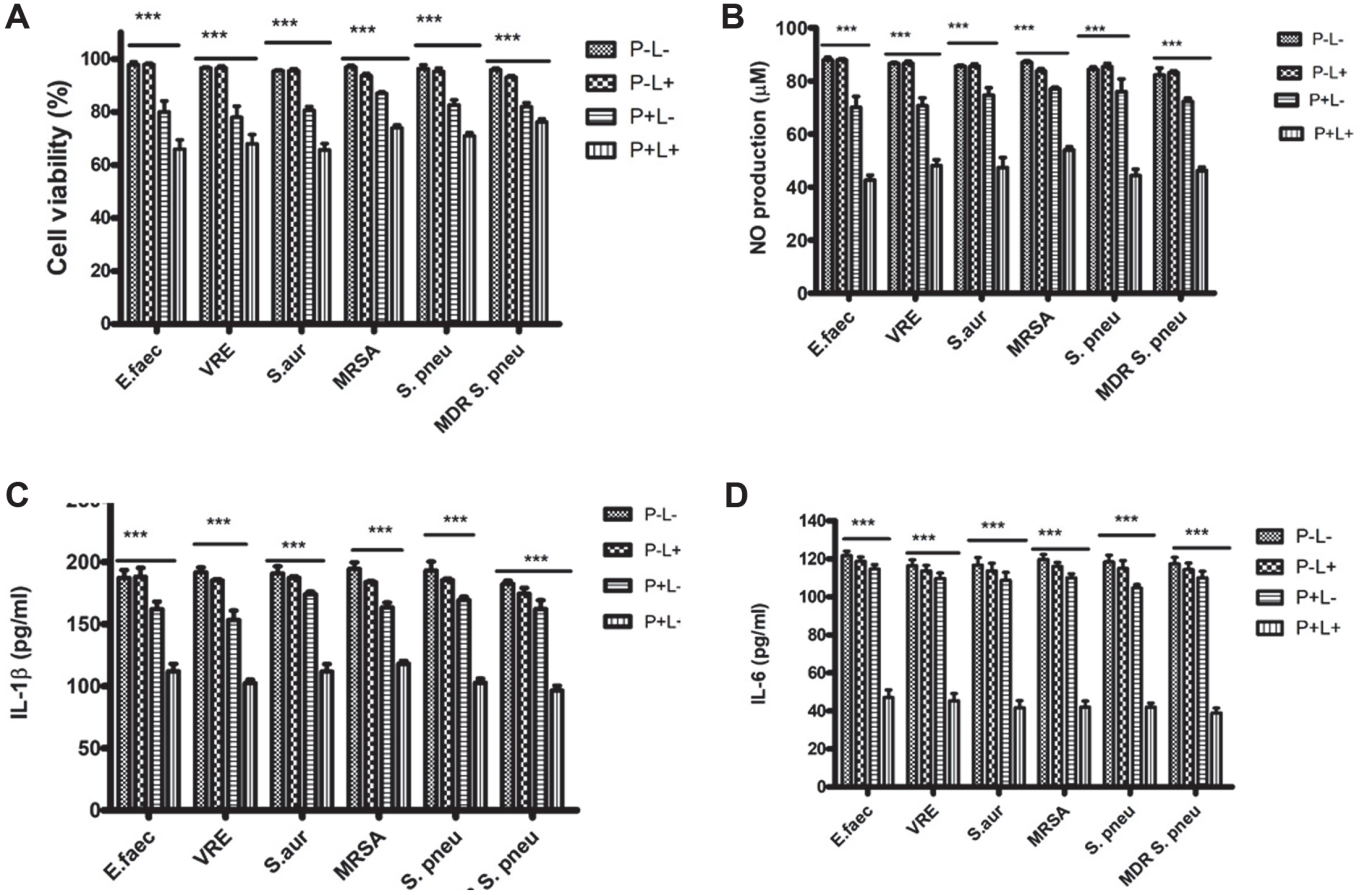
A-D: Effects of AE-mediated PDT on viability and pro-inflammation factors production in bacterialinfected RAW264.7 cells. A: RAW264.7 cells infected with *E. faecalis* (ATCC 29212), VRE (ATCC 51299), *S. aureus* (ATCC 29213), MRSA, *S. pneumoniae* (ATCC 49619), and MDR *S. pneumoniae* (ATCC 49619), were plated in 96-wells plates and treated with either light (72 J/cm^2^) (P+L-), AE (32 μg/ml) alone (P+L-) or both (P+L+) or no treatment (P-L-) for 24 h. A CCK- 8 assay determined the proliferation, and expression was done relative to control (DMSO). B: Bacteria-infected RAW264.7 cells were seeded in 24-wells culture plates overnight and treated with P-L-, P-L+, P+L- or P+L+. NO levels in the culture media were determined using Griess reagent. C and D: Bacteria-infected RAW264.7 cells plated in 24-well culture plates overnight were pretreated with P-L+, P+L-, or P+L+ and cultured for 24 h. The IL-1β and IL-6 concentration was determined by ELISA. The experiments were conducted thrice in triplicates, and data were analyzed through two-way ANOVA. ***p<0.001 compared to no treatment (P-L-).

Cell viability studies result demonstrated significantly inhibited growth when the infected RAW264.7 cells were treated with a combination of AE (of 32 μg/ml) and light (900 s), compared to the controls (data not shown) or single treatments (Fig. 5A). NO is a well-described inflammatory response marker. Griess reagent reaction was used to determine the effect of treatment on NO production. Bacterial infections significantly stimulated the production of NO (data not shown), and the treatment with both AE and light significantly downregulated NO generation compared to the single treatment groups (Fig. 5B).

Further, we investigated whether AE-PDT affects pro-inflammatory cytokine production in bacteria-infected RAW264.7 cells. As demonstrated in Fig. 5C, AE-PDT significantly repressed IL-1β production compared to the bacteria-infected cells treated with either light or AE alone. Similarly, IL-6 production was significantly suppressed following the treatment of the cells with AE-PDT compared to the cells singly treated with light or AE (Fig. 5D).

**Fig. 6:**
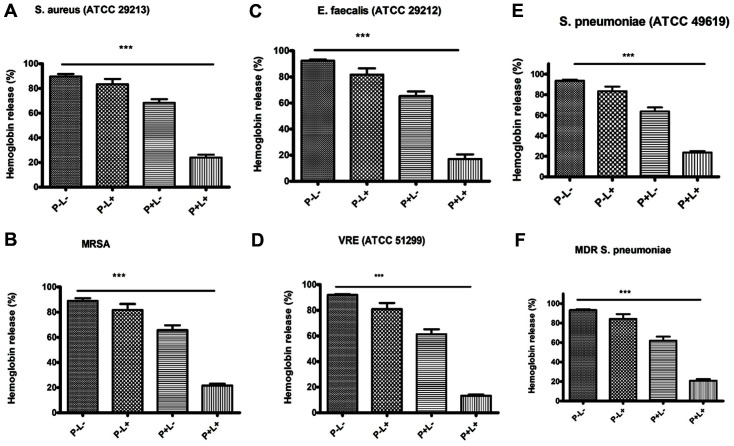
A-F the indicated gram-positive bacteria were cultured with various treatments; negative control (PL-), AE alone (32 μg/ml) (P+L-), light alone (72 J/cm^2^) (P-L+), AE and light (P+L+). The supernatants were then used to assess the hemolytic activity. The observed hemolytic activity of culture bacteria supernatants co-cultured with various treatments were **A**: *S. aureus* (ATCC 29213) 89.67% (P-L-) 83.33 % (P-L+) 68.33 % (P+L-) and 24% (P+L+) **B**: MRSA; 89% (PL-) 81.67% (P-L+) 65.67% (P+L-) and 21.67% (P+L+) **C**: *E. faecalis* (ATCC 29212) 92.33% (P-L-) , 81.67% (P-L+) 65.33% (P+L-) and 17 % (P+L+) **D**: VRE (ATCC 51299): 93% (P-L-) 81% (P-L+) 61.33% (P+L-) and 13.3% (P+L+) **E**: *S. pneumoniae* (ATCC 49619); 93.67% (P-L-) 83.33% (P-L+) 63.67% (P+L-) and 23.66% (P+L+). **F**: MDR-S pneumoniae: 93.33% (P-L-) 84.33% (P-L+) 62% (P+L-) and 21% (P+L+). ***Shows *p* < 0.0001, compared with the untreated (P-L-) group; two-tailed *t*-test.

Next, we investigated the potential AE-mediated photodynamic inactivation suppressor effects against cytolysin, d-hemolysin, and pneumolysin. The hemolytic activity of the toxins was assessed in post-bacterial treatments with light or AE alone or a combination of both. According to the observations, an insignificant reduction in the hemolytic activity was reported following treatment with light or AE alone. However, after treatment with AE and irradiation, the hemolytic activity was significantly decreased to 17% (*E. faecalis*), 13.3%(VRE), 24% (*S. aureus*), 21.67% (MRSA), 23.66% (*S. pneumoniae*), and 21% (MDR *S. pneumoniae*). In the control groups without AE or light treatment, δ-hemolysin lysed 89.67% and 89% of rabbit erythrocytes in *S. aureus* and MRSA, respectively. Cytolysin toxin lysed 92.33% and 93% of the erythrocytes in *E. faecalis* and VRE, respectively, while pneumolysin lysed 93.67% and 93.33% erythrocytes in *S. pneumoniae* and MDR *S. pneumoniae*, respectively (Figs. 6A-6F).

**Fig. 7:**
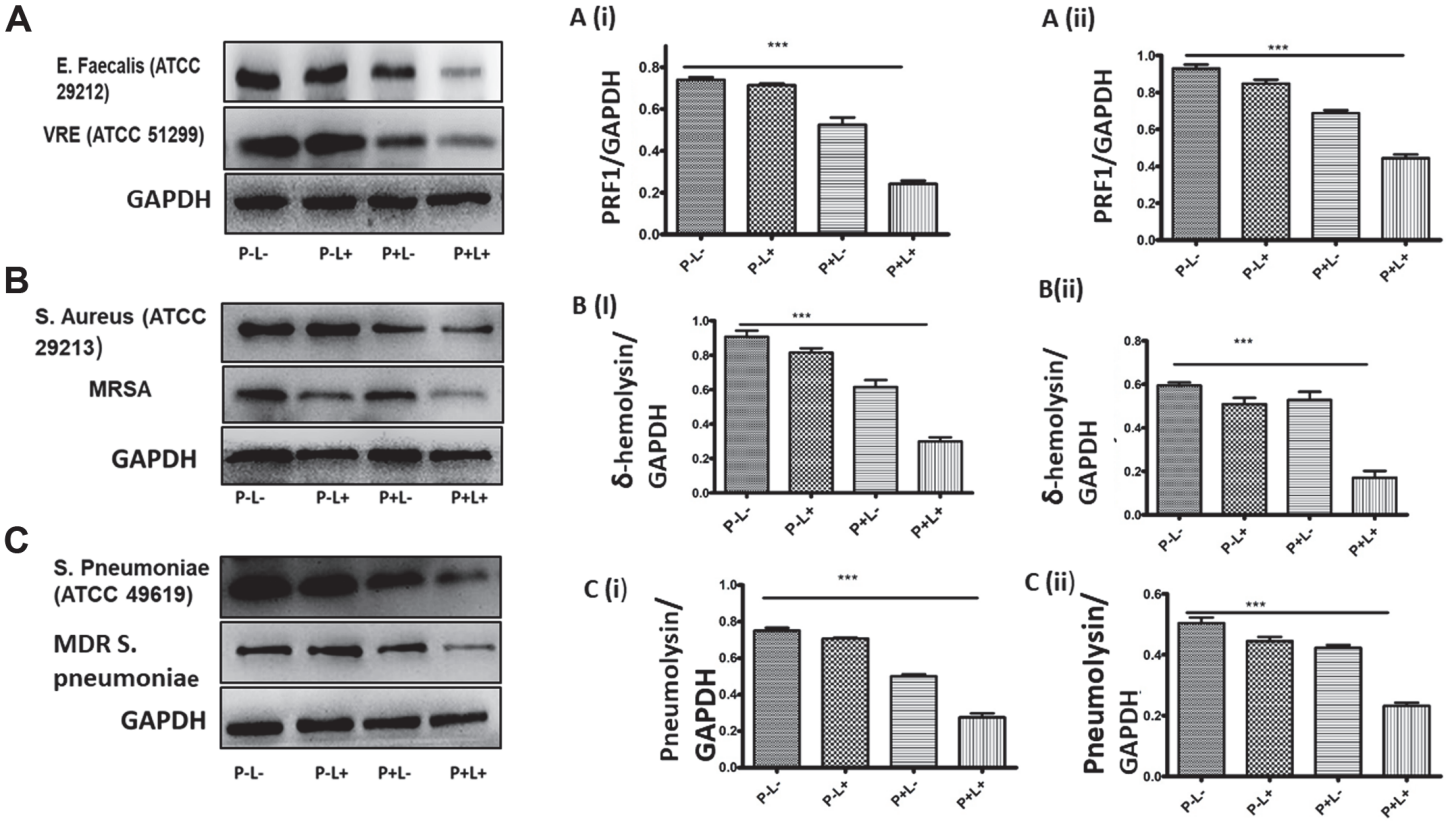
Western blot assays determining the expressions of sepsis-associated exotoxins following bacterial treatments with light alone (72 J/cm^2^) (P-L+), AE (32 μg/ml) alone (P+L-) or AE combined with light (P+L+) or no treatment (P-L-). **A**: Cytolysin toxin expression in *Enterococcus* spp. Following various treatments as stated. **A (i)**: Bar graph representation of cytolysin toxin in *E. faecalis* (ATCC 29212) and **A (ii)**: Bar graph representation of cytolysin toxin in VRE ATCC 51299). **B**: δ-hemolysin toxin expression in *Staphylococcus* spp. following various treatments as stated. **B (i)**: Bar graph representation of δ-hemolysin in *S. aureus* (ATCC 29213). **B (ii)**: Bar graph representation of δ-hemolysin in MRSA. **C**: Pneumolysin toxin expression in *Streptococcus* spp. following various treatments as stated. **C (i)**: Bar graph representation of pneumolysin in *S. pneumoniae* (ATCC 49619). **C (ii)**: Bar graph representation of pneumolysin in MDR *S. pneumoniae*. GAPDH was used as the internal control. ***Indicates *p* < 0.0001 compared with the untreated control group in a two-tailed Student’s *t*-test.

Finally, we assessed whether AE-mediated photodynamic inactivation interferes with expression of cytolysin, δ-hemolysin, and pneumolysin, which are the toxins responsible for the pathogenicity of the studied gram-positive bacteria. According to the western blot results, a single treatment with either AE or light or lack of it, did not significantly interfere with PRF1 expression in both *E. faecalis* and VRE. However, the AE-mediated photodynamic inactivation significantly suppressed PRF1 expression in *E. faecalis* and VRE, as shown in Figs. 7A (i-ii) (*p* > 0.0001).

For the single treatments in *S. aureus* and MRSA, δ-hemolysin expression was not significantly reduced, while AE-PDT drastically reduced its expression as shown in Figs. 7B (i) and 7B (ii), respectively (*p* > 0.0001). Similarly, single treatments or negative control studies did not demonstrate any significant reduction in pneumolysin expression in *S. pneumoniae* and MDR *S. pneumoniae*. Nevertheless, AE-mediated PDT significantly inhibited its expression, as depicted in Figs. 7C (i) and 7C (ii), respectively (*p* > 0.001).

## Discussion

Persistent antimicrobials misuse has gradually led to their resistance, consequently leading to discontinuation after decades of use and the occurrence of various multidrug-resistant bacterial strains such as vancomycin-resistant *Enterococcus*, MRSA, and MDR *S. pneumoniae* [16, 17]. Antibiotics use is directly associated with the survival of bacteria and results in resistant strains selection, finally to resistant flora. Therefore, there is a possibility that various antimicrobial drugs may finally cause resistance to drugs because of the excellent environmental adaptation capability of bacteria. Consequently, new and effective alternative treatment approaches are necessary to address the challenges of resistance.

According to multiple studies, targeting virulence factors is a better strategy that has demonstrated significant results [18, 19]. Gram-positive bacteria generally induce inflammation by releasing cytolytic exotoxins, which are significant virulence factors that destroy the host’s cell membrane and initiate various inflammatory processes [20]. PDT has been widely discussed as a promising tolerable and affordable alternative for treating bacterial infections [21]. However, investigations on its effects on gram-positive bacteria toxins do not exist.

The current study reports the photodynamic effects of AE coupled with light (435 ± 10 nm) against *Enterococcus*, *Staphylococcus*, and streptococcal pathogens, which occur in an AE-dose-dependent manner. AE (2-4 μg/ml) and light irradiation did not inactivate the bacteria. However, 8, 16, and 32 μg/ml and irradiation all had reduced effects on the survival of bacteria in an AE-dose dependent manner with over 7 log10 bacteria survival reduction in all the strains tested when 32 μg/ml AE was used.

Biofilms ensure microbes are protected from antibiotics and the host’s immunity [22]. Biofilms of bacteria have been linked with infections such as periodontitis, cystic fibrosis, and nosocomial diseases on heart valves and catheters [23, 24]. Our findings reported biofilm formation in all the bacteria under study. This observation is in agreement with a previous report that most isolates of *Enterococcus*, *Staphylococcus*, and *Streptococcus* bacteria can form biofilms and are therefore the reason for their resistance to at least one antibiotic [25].

Because of the role of biofilm formations in the successful establishment of infection, its destruction is an important way of curbing gram-positive bacterial infections. PDT has been shown to reduce the formation of biofilm in various studies [26]. We have also shown that PDT reduces the formation of biofilms following the use of Hypocrellin B and light [27]. This study further confirmed that AE-mediated PDT also reduced the formation of biofilms in gram-positive bacteria.

This study also reported an inhibited NO, IL-1β, and IL-6 production in RAW264.7 cells, confirming the anti-inflammatory potency of AE-mediated PDT. NO is an important pro-inflammatory cytokine for various infections, and its regulation is important for the control of sepsis [28]. Nitric oxide is generated in increased levels by iNOS proteins stimulated by pathogenic products, for instance, LPS. IL-1β is regarded as an early-produced pro-inflammatory cytokine that induces inflammation at the local and systemic levels [29]. IL-6 also plays a critical function in various inflammatory conditions, particularly in the acute-phase reactions [30].

Progressed gram-positive bacterial infections often lead to sepsis, whose hallmark is the presence of at least two systemic inflammation features, such as hypothermia or fever, leukopenia or leukocytosis, tachypnea, and tachycardia [31]. Enterococcal septicemia mediates an inflammatory severe immune response, which is a predisposing factor to secondary bacterial infections. The result is an increased septic shock incidence and multiple organ failure, eventually leading to increased mortality [32]. *Staphylococcus* spp. is associated with toxic shock syndrome (TSS), characterized by hypotension, organs failure, rash, fever, and myalgia [1]. Besides, *S. pneumoniae* has been linked with sepsis, referred to as invasive pneumococcal disease (IPD) [33]. Amongst various exotoxins produced by *Enterococcus* spp., cytolysin is a hemolytic and bactericidal toxin that causes multiple organ failure and death [34]. *Staphylococcus* spp., also produce δ-hemolysin, which remains a well-characterized and prominent hemolytic exotoxin responsible for the pathogenesis of staphylococcal infections, killing various populations of host cells, such as the immune cells, and assisting in the spread of the pathogen within the host [35]. In *S. pneumoniae*, pneumolysin (PLY) is a hemolytic exotoxin essential in various pneumococcal disease steps. PLY cooperates with invasins, proteases, and adhesins for successful pathogenesis. PLY consequently contributes to the penetration of bacteria and inflammation, leading to the direct destruction of cells by a pore-forming cytolysis process and abetting the escape of bacteria through complement system blocking [36]. Indeed, our findings confirmed the hemolytic activities of cytolysin, δ-hemolysin, and PLY, which were significantly reduced following AE-mediated PDT. The expressions of these exotoxins were also significantly reduced following AE-mediated PDT as was denoted by inhibited PRF1, δ-hemolysin, and pneumolysin in the *Enterococcus* spp., *Staphylococcus* spp., and the *Streptococcus* spp., studied.

## Conclusion

In conclusion, our findings confirmed that combined use of AE and light (435 ± 10 nm) inactivates MRSA, *S. aureus* (ATCC 29213), *S. pneumoniae* (ATCC 49619), MDR-*S. pneumoniae*, *E. faecalis* (ATCC 29212), and VRE (ATCC 51299) in an AE-dose dependent manner. AE and light are also effective in reducing biofilm formations, suppressing NO, IL-1β, IL-6, hemolytic activities, and inhibiting the expressions of toxins that cause sepsis.
